# NPAS2 attenuates diabetic cardiomyopathy by preserving pyruvate dehydrogenase activity and mitochondrial metabolic homeostasis

**DOI:** 10.3389/fcvm.2026.1980726

**Published:** 2026-10-19

**Authors:** Wenjiao Li, Bin Han, Di Zhu, Lixia Luan, Xuyun Dang

**Affiliations:** 1 Department of Anesthesiology, The First Affiliated Hospital of Xi’an Medical University, Xi’an, China; 2 Chang’an University Hospital, Chang’an University, Xi’an, China

**Keywords:** circadian rhythm, diabetic cardiomyopathy, glucose intolerance, mitochondrial metabolism, NPAS2, pyruvate dehydrogenase

## Abstract

**Background:**

Diabetic cardiomyopathy (DCM) is a serious cardiac complication of diabetes, characterized by myocardial metabolic dysregulation, mitochondrial dysfunction, and structural remodeling. Disruption of circadian clock genes has been implicated in metabolic diseases, yet the role of neuronal PAS domain protein 2 (NPAS2), a core circadian regulator, in DCM remains poorly understood.

**Methods:**

NPAS2 expression and circadian rhythmicity were assessed in the myocardium of T2DM and *db/db* mice. Cardiac-specific *Npas2* knockout (*Npas2*^cko^) mice were subjected to high-fat diet combined with streptozotocin (HFD + STZ) to model DCM and evaluate cardiac function, remodeling, and metabolic profiles. H9c2 cardiomyocytes were used for loss-of-function and gain-of-function experiments under high glucose/high fat (HGHF) conditions, and rescue experiments targeting pyruvate dehydrogenase (PDH) were performed to establish downstream mechanisms. Finally, adeno-associated virus serotype 9 (AAV9) mediated NPAS2 overexpression was delivered *in vivo* to evaluate its therapeutic potential.

**Results:**

Myocardial NPAS2 expression was significantly reduced and its circadian oscillations were disrupted in diabetic mice. Cardiomyocyte-specific *Npas2* deletion exacerbated HFD + STZ-induced cardiac dysfunction, hypertrophy, fibrosis, and lipid accumulation. In H9c2 cells, NPAS2 knockdown impaired mitochondrial respiration, increased apoptosis, elevated triglyceride and free fatty acid contents, and reduced PDH activity. Conversely, NPAS2 overexpression reversed these detrimental effects under HGHF conditions. Rescue experiments demonstrated that PDH overexpression mitigated the damage caused by NPAS2 knockdown, whereas PDH silencing abolished the protective effects of NPAS2 overexpression. *In vivo*, AAV9-mediated NPAS2 overexpression significantly improved cardiac function, attenuated hypertrophy and fibrosis, and restored lipid and glucose homeostasis in diabetic WT and *Npas2*^cko^ mice.

**Conclusion:**

NPAS2 protects the heart against diabetic cardiomyopathy by maintaining PDH-dependent mitochondrial oxidative metabolism and metabolic homeostasis, representing a promising therapeutic target for DCM.

## 1 Introduction

Diabetic cardiomyopathy (DCM) is a distinct cardiac complication of diabetes mellitus, characterized by left ventricular diastolic and systolic dysfunction, cardiomyocyte hypertrophy, interstitial fibrosis, and myocardial metabolic remodeling, independent of coronary artery disease and hypertension (1). With the rapidly increasing global prevalence of diabetes, DCM has become a leading contributor to heart failure and cardiovascular mortality in diabetic patients (2). Although the pathogenesis of DCM is multifactorial, growing evidence has established that impaired myocardial substrate metabolism, mitochondrial dysfunction, excessive oxidative stress, and cardiomyocyte apoptosis are central events driving disease initiation and progression (3). Among these abnormalities, the shift in cardiac energy substrate preference from glucose oxidation toward fatty acid utilization plays a particularly critical role, leading to lipotoxicity, mitochondrial inefficiency, and ultimately contractile dysfunction (4, 5). Nevertheless, the upstream regulatory mechanisms that coordinate circadian signals with myocardial energy metabolism in the diabetic heart remain largely unknown.

The circadian clock is an evolutionarily conserved molecular system that generates endogenous rhythms in gene expression, metabolic flux, and physiological function across nearly all mammalian tissues (6). In mammals, the core molecular clock is composed of at least ten identified circadian genes, including *Bmal1, Clock, Npas2, Per1/2/3, Cry1/2, Tim*, and *Cki*, which form interlocking transcription–translation feedback loops to regulate daily rhythms (7). Accumulating evidence indicates that disruption of circadian rhythms is closely associated with the pathogenesis of metabolic diseases, including obesity, type 2 diabetes, dyslipidemia, and cardiovascular disorders (7, 8). These effects are mediated, at least in part, through circadian control of metabolic gene expression and mitochondrial function (6).

Among the core clock components, neuronal PAS domain protein 2 (NPAS2) has emerged as a particularly important regulator of cellular metabolism and stress responses (9). NPAS2 is widely expressed in mammalian tissues and predominantly localized in the nucleus, where it heterodimerizes with BMAL1 to drive the transcription of clock-controlled genes. Beyond its canonical role in circadian rhythm generation, NPAS2 participates in diverse biological processes, including cell cycle regulation, DNA damage repair, hormone secretion, immune responses, inflammatory signaling, and energy metabolism. Previous studies have reported that NPAS2 expression is altered in various pathological conditions, including cardiovascular disease, rheumatoid arthritis, Alzheimer’s disease, Parkinson’s disease, and multiple types of cancer (10–12). In the cardiovascular system, NPAS2 expression was found to be progressively downregulated during myocardial ischemia/reperfusion injury, and overexpression of NPAS2 markedly attenuated cardiomyocyte apoptosis and improved cardiac function, suggesting a protective role of NPAS2 against cardiac stress (10). In addition, NPAS2 has been implicated in the regulation of blood pressure and the pathogenesis of hypertension (13, 14). These findings highlight NPAS2 as a promising cardioprotective factor, yet its specific role in the diabetic myocardium has not been systematically explored.

Emerging evidence has further linked NPAS2 to the regulation of glucose and lipid metabolism. High-fat diet feeding has been shown to alter NPAS2 expression in skeletal muscle, suggesting a reciprocal relationship between nutritional status and this circadian regulator (15). Notably, NPAS2 deficiency in the liver results in severe hepatic steatosis and dysregulation of lipoprotein metabolism, underscoring the essential role of NPAS2 in maintaining lipid homeostasis (16). Conversely, NPAS2 is upregulated in hepatocellular carcinoma, where it promotes glycolysis by increasing the expression of glycolytic genes such as GLUT1, PKM2, and MCP4, while simultaneously suppressing mitochondrial biogenesis and oxidative phosphorylation through downregulation of PGC-1*α* (17). These observations demonstrate that NPAS2 can exert tissue-specific metabolic effects, and its functional output is likely dependent on the cellular context. However, whether NPAS2 directly regulates myocardial glucose and lipid metabolism in the setting of diabetic cardiomyopathy has not been previously reported.

Pyruvate dehydrogenase (PDH) is the rate-limiting enzyme of the pyruvate dehydrogenase complex (PDHc), which catalyzes the irreversible oxidative decarboxylation of pyruvate to acetyl-CoA, thereby linking glycolysis to the tricarboxylic acid cycle and oxidative phosphorylation. PDH activity is a critical determinant of myocardial glucose oxidation, and its suppression in the diabetic heart leads to reduced glucose utilization, increased fatty acid oxidation, mitochondrial dysfunction, and aggravated cardiac injury (18). Studies have demonstrated that enhancing PDH activity protects the heart against ischemia/reperfusion injury and improves myocardial substrate utilization (19). Moreover, in diabetic cardiomyopathy, PDH activity is significantly reduced, contributing to the metabolic inflexibility and lipotoxic injury that characterize the diabetic heart (20). Interestingly, previous work has revealed that muscle-specific deletion of the circadian gene Bmal1 impairs insulin-stimulated glucose uptake in skeletal muscle, accompanied by a significant reduction in PDH activity, indicating an intimate functional link between circadian clock components and PDH-mediated glucose oxidation (21). Nevertheless, whether NPAS2 governs PDH expression or activity in the heart, and whether such regulation is mechanistically relevant to diabetic cardiomyopathy, remains entirely unknown.

In the present study, we hypothesized that NPAS2 serves as a critical upstream regulator of myocardial PDH activity and glucose oxidation, and that disruption of NPAS2 contributes to the metabolic and structural abnormalities observed in diabetic cardiomyopathy. To test this hypothesis, we first characterized the expression and circadian rhythmicity of NPAS2 in the myocardium of two independent diabetic mouse models. We then examined the functional consequences of cardiomyocyte-specific *Npas2* deletion under diabetic conditions *in vivo*, and dissected the underlying cellular and molecular mechanisms using H9c2 cardiomyocytes subjected to high-glucose/high-fat stress. Finally, we evaluated the therapeutic potential of adeno-associated virus serotype 9 (AAV9) mediated NPAS2 overexpression in diabetic mice. Our results demonstrate that NPAS2 is a pivotal endogenous protector of the diabetic heart, acting through PDH-dependent preservation of mitochondrial oxidative metabolism and cardiac metabolic homeostasis. These findings identify the NPAS2–PDH axis as a previously unrecognized signaling pathway with significant translational implications for the prevention and treatment of diabetic cardiomyopathy.

## 2 Method

### 2.1 Animal models

All male C57BL/6J mice were obtained from the Animal Center of Xi’an Medical University. Receptor-deficient C57BLKS (*db/db*) mice and wild-type C57BLKS mice were purchased from Beijing Vital River Laboratory Animal Technology Co., Ltd. (Beijing, China). *Npas2*^flox/flox^ mice on a C57BL/6 background were generated by Nanfang Biotech Technology Co., Ltd. (Shanghai, China). Cardiomyocyte-specific *Npas2* knockout mice (*Npas2*^cko^) were generated by crossing Myh6-Cre transgenic mice with *Npsa2*^flox/flox^ mice and inducing recombination by intraperitoneal administration of tamoxifen (TMX, 50 mg/kg) for five consecutive days. *Npsa2*^flox/flox^ littermates receiving TMX served as controls. All animal procedures were conducted in accordance with the Guidelines for Animal Protection and Use of the People’s Republic of China and were approved by the Ethics Committee of First Affiliated Hospital of Xi’an Medical University (Ethical Number: XYYFY2025LSDW-002). All mice were housed in the Animal Experiment Center of Xi’an Medical University under specific pathogen-free conditions at 20–23℃ with a 12 h light/dark cycle. The light was switched on from 6:00 a.m. (zeitgeber time 0, ZT0) to 6:00 p.m. (ZT12), defining the light phase as ZT0–ZT12 and the dark phase as ZT12–ZT24. Experimental animals were randomly assigned to groups, and investigators were blinded to group allocation during *in vivo* experiments and subsequent analyses.

### 2.2 Adeno-associated virus injection

Recombinant adeno-associated virus serotype 9 (AAV9) was diluted in sterile phosphate-buffered saline (PBS) to a final titer of 2 × 10^13^ vector genomes (vg)/mL. At 9 weeks of age, male mice received intravenous injection via the lateral tail vein under brief isoflurane anesthesia (2% for induction). A total volume of 25 μL viral suspension was administered using a 30-gauge needle. Hemostasis was achieved by gentle compression, and mice were monitored until full recovery. Animals were maintained for an additional 2 weeks to allow transgene expression. Viral transduction efficiency was confirmed postmortem in pilot animals by Western blot analysis.

### 2.3 Experimental mouse diabetes model

Male mice at 4 weeks of age were fed a high-fat diet (HFD, 45% kcal from fat) for 4 weeks, followed by intraperitoneal injection of streptozotocin. Streptozotocin (STZ, 100 mg/kg body weight; Sigma-Aldrich, USA, catalog No. S0130). STZ was freshly dissolved in prechilled citric acid buffer (pH 4.2–4.5) at 4℃, protected from light, and administered within 30 min after preparation. Blood glucose levels ≥16.7 mmol/L at 48 h after injection were considered indicative of successful diabetes induction. Control mice received an equivalent volume of citric acid buffer after fasting. At this stage, mice were approximately 8–9 weeks old and continued on the HFD.

For cardiac-specific NPAS2 overexpression, adeno-associated virus serotype 9 carrying the cardiomyocyte-specific cTNT promoter-driven 3Flag-NPAS2 construct (AAV9-cTNT-NPAS2) and control virus (AAV9-cTNT-Ctrl) were obtained from GeneChem Co., Ltd. (Shanghai, China). Diabetic mice at 9 weeks of age were injected via the tail vein with AAV9-cTNT-Ctrl or AAV9-cTNT-NPAS2. Cardiac function in 16-week-old mice was assessed by echocardiography. Masson’s trichrome staining and wheat germ agglutinin (WGA) staining were performed to evaluate pathological alterations in 16-week-old *Npsa2*^cko^ mice. Intraperitoneal glucose tolerance tests (IPGTTs) were performed as described previously (22).

### 2.4 Evaluation of echocardiography

As described previously (23), cardiac function was evaluated using a Vevo 2100 ultrasound system (VisualSonics, Toronto, ON, Canada). Briefly, mice were lightly anesthetized with isoflurane (3% for induction and 1% for maintenance), and echocardiographic images were acquired using a hand-held ultrasound transducer. Following image acquisition, heart rate (HR), left ventricular ejection fraction (LVEF), left ventricular fractional shortening (LVFS), and interventricular septal (IVS) thickness were analyzed using Vevo 2100 software. All measurements were performed by a blinded investigator.

### 2.5 Histological and immunofluorescence analysis

Mouse hearts were fixed in 4% paraformaldehyde for 48 h, embedded in paraffin, and sectioned according to standard procedures. Masson’s trichrome staining was performed according to the manufacturer’s instructions (Solarbio, Beijing, China, Cat. No. G1346). Cardiomyocyte cross-sectional area was assessed by FITC-conjugated wheat germ agglutinin (WGA) staining according to the manufacturer’s protocol (Sigma-Aldrich, USA, Cat. No. L4895). Immunohistochemical staining for NPAS2 was performed on heart tissue sections using a primary anti-NPAS2 antibody (Novus Biologicals, Centennial, CO, USA, Cat. No. NBP1-88612). Tissue sections were cut into 3–5 μm slices, and images were acquired with a Nikon Eclipse Ni-U microscope at × 400 magnification. Six fields were randomly selected per section for analysis.

### 2.6 Western blotting

Protein lysates from left ventricular tissue were prepared in RIPA buffer supplemented with protease and phosphatase inhibitors. Equal amounts of protein (30 μg per lane) were separated by 10% SDS-PAGE and transferred to PVDF membranes. The membranes were incubated with the following primary antibodies: anti-NPAS2 primary antibody (Thermo Fisher Scientific, USA, Cat. No. PA5-75540, 1:1000 dilution for Western blot). and anti-GAPDH primary antibody (Proteintech, Wuhan, China, Cat. No. 60004-1-Ig, 1:10000 dilution for Western blot). After incubation with HRP-conjugated secondary antibodies (1:5000), signals were detected using an ECL substrate (Bio-Rad) and quantified with Image Lab v6.0 software. All compared samples were resolved on the same gel, transferred to the same membrane, and processed simultaneously. Protein expression was normalized to GAPDH, and only signals within the linear detection range were included in the analysis.

### 2.7 qRT-PCR analysis

Total RNA was extracted from left ventricular tissues using Trizol reagent. Complementary DNA was synthesized using the PrimeScript RT reagent kit and subsequently amplified using SYBR Green Master Mix. The primer sequences were as follows: *Npsa2* forward: 5'- ACAGCACCACAGCTTTGCCAAG-3’, *Npsa2* reverse: 5'- CAGCAGGAGTTGCTTTGTGAGG-3’, GAPDH forward: 5'- CATCACTGCCACCCAGAAGACTG-3’, and GAPDH reverse: 5'- ATGCCAGTGAGCTTCCCGTTCAG-3’. Relative mRNA expression was calculated using the 2^(−*ΔΔ*Ct)^ method with GAPDH as the internal control. Data are presented as fold change relative to the sham or control group.

### 2.8 TG and FFA contents

At the end of the intervention, cardiac tissues were harvested and immediately snap-frozen in liquid nitrogen. Triglyceride (TG) and free fatty acid (FFA) contents in heart tissues and H9c2 cells were measured using a Triglyceride (TG) assay kit (Cat. No. A110-1-1; Nanjing Jiancheng Bioengineering Institute, Nanjing, Jiangsu, China) and a Free fatty acid (FFA) assay kit (Cat. No. ab65341; Abcam, Cambridge, UK), respectively, according to the manufacturer’s protocols. All measurements were normalized to protein concentration.

### 2.9 Cell culture

H9c2 rat cardiomyocytes were obtained from Servicebio (Wuhan, China) and cultured in DMEM supplemented with 10% fetal bovine serum and 1% penicillin/streptomycin. To mimic the diabetic microenvironment, cells were treated with DMEM containing 33 mM glucose and 100 μM palmitate (Sigma-Aldrich, St. Louis, MO, USA; catalog No. P9767) for 24 h, as previously described (24).

### 2.10. SiRNA transfection and adenoviral overexpression

*Npsa2*-targeting siRNA, PDH-targeting siRNA, and negative control siRNA were synthesized by Qingke Biotechnology (Xi’an, China). siRNA was diluted to a final concentration of 20 nM and mixed with RNA iMAX in OptiMEM at a 1:50 ratio, followed by incubation for 20 min at room temperature. The complexes were then added to cells and incubated for 48 h. For adenoviral overexpression, Ad-NPAS2, Ad-PDH, and Ad-EV were purchased from HanBio (Shanghai, China). Cells were infected at a multiplicity of infection (MOI) of 50 in serum-free medium for 6 h, after which the medium was replaced with complete medium for an additional 42 h, yielding a total incubation time of 48 h.

### 2.11. Mitochondrial function assays

Oxygen consumption rate (OCR) weas measured using the Seahorse XFe96 Analyzer (Agilent Technologies, Santa Clara, CA, USA). Seahorse XFe96/XF Pro FluxPak consumables (Cat. No. 103792-100; Agilent Technologies, Santa Clara, CA, USA) were used for the assay. H9c2 cells were seeded in XF96 plates at a density of 20,000 cells per well. Before measurement, cells were incubated in assay medium consisting of XF base medium supplemented with 10 mM glucose, 1 mM pyruvate, and 2 mM glutamine (pH 7.4) for 1 h at 37 °C in a non-CO_2_ incubator. Basal OCR was recorded over three measurement cycles consisting of mixing for 3 min, waiting for 2 min, and measuring for 3 min. After baseline measurements, oligomycin (final concentration, 1 μM) was sequentially injected to inhibit ATP synthase and assess ATP-linked respiration, followed by FCCP (0.5 μM) to uncouple electron transport and determine maximal respiratory capacity. Finally, rotenone/antimycin A (0.5 μM) was added to inhibit complexes I and III, thereby defining non-mitochondrial respiration.

### 2.12. Apoptosis analysis via annexin V/PI staining

Apoptosis in H9c2 cells was quantified using a FITC Annexin V Apoptosis Detection Kit (Cat. No. C1062S; Beyotime Biotechnology, Shanghai, China). After treatment, cells were washed twice with ice-cold PBS and resuspended in Annexin V binding buffer at a concentration of 1 × 10^6^ cells/mL. A total of 100 μL cell suspension was incubated with 5 μL Annexin V-FITC for 15 min at room temperature in the dark, followed by addition of 5 μL propidium iodide immediately before analysis. Samples were analyzed using a BD FACSCanto II flow cytometer with 488-nm excitation. At least 10,000 events were recorded for each sample.

### 2.13. Extracellular pyruvate and lactic acid assays

Pyruvate and lactic acid contents were measured using the pyruvate assay kit (Cat. No. A081-1-1) and lactic acid assay kit (Cat. No. A019-2-1) from Nanjing Jiancheng Bioengineering Institute (Nanjing, Jiangsu, China), following the manufacturer’s protocols. Absorbance values were read by a microplate reader, and metabolite levels were normalized to the total protein concentration of each sample.

### 2.14. Measurement of pyruvate dehydrogenase (PDH) activity

PDH activity was determined using a Pyruvate Dehydrogenase Activity Colorimetric Assay Kit (Cat. No. ab287837; Abcam, Cambridge, UK) according to the manufacturer’s instructions. The assay is based on the catalytic conversion of pyruvate to acetyl-CoA by PDH, which generates a reaction intermediate that reduces the developer to a colored product measurable at 450 nm. PDH activity was calculated from the NADH standard curve and expressed as mU/mL.

### 2.15. Malondialdehyde (MDA) content

MDA levels in cardiomyocytes were measured using an MDA assay kit (Cat. No. MAK085-1KT; Sigma-Aldrich, St. Louis, MO, USA). Briefly, cells subjected to different treatments were homogenized and mixed with thiobarbituric acid reagent. The mixture was boiled for 15 min and then centrifuged at 4000 rpm for 8 min. MDA content was determined by measuring absorbance at 532 nm.

### 2.16. Statistical analysis

Statistical analyses were performed using SPSS 23.0. Continuous variables with a normal distribution are presented as the mean ± standard deviation (SD). Normality was assessed using the Shapiro–Wilk test, and all datasets met the assumption of normality. Homogeneity of variance was examined using Bartlett’s test. Comparisons between two groups were made using the unpaired Student’s t-test. One-way ANOVA was used for comparisons among multiple groups involving a single factor, whereas two-way ANOVA was applied when two independent factors were present, followed by Bonferroni *post hoc* tests. For repeated-measures data, two-way repeated-measures ANOVA was used. GraphPad Prism 8.3.0 (GraphPad Software, Inc., USA) was used for data visualization and statistical analysis. *P* < 0.05 was considered statistically significant.

## 3 Results

### 3.1 NPAS2 expression is downregulated and exhibits circadian rhythm disruption in the myocardium of diabetic mice

To characterize the expression profile of NPAS2 in diabetic cardiomyopathy, we first measured myocardial NPAS2 mRNA expression at six-hour intervals over a 24-hour period (ZT0, ZT6, ZT12, ZT18, and ZT24) in T2DM mice, *db/db* mice, and their corresponding control mice. Mice were maintained under a 12 h light/12 h dark cycle (light on at 6:00 a.m., defined as ZT0; light off at 6:00 p.m., defined as ZT12). Compared with control mice, diabetic mice exhibited significantly reduced NPAS2 mRNA levels in the myocardium, accompanied by disrupted circadian rhythmicity (Figure 1A). Given that the most prominent mRNA changes were observed at ZT0 and ZT12, we next assessed NPAS2 protein expression at these two time points. Consistent with the mRNA findings, NPAS2 protein levels were markedly decreased in both T2DM and *db/db* mice when compared with normal controls at ZT0 and ZT12 (Figures 1B,C). Furthermore, immunohistochemical analysis was performed to evaluate myocardial NPAS2 expression in T2DM mice at 8, 12, and 16 weeks of age. Notably, NPAS2 expression in the myocardium of diabetic mice was already significantly reduced at 8 weeks of age relative to age-matched controls, and this reduction became more pronounced with advancing age (Figures 1D,E). Together, these findings demonstrate that NPAS2 expression is suppressed and its circadian oscillation is perturbed in the diabetic heart.

**Figure 1:**
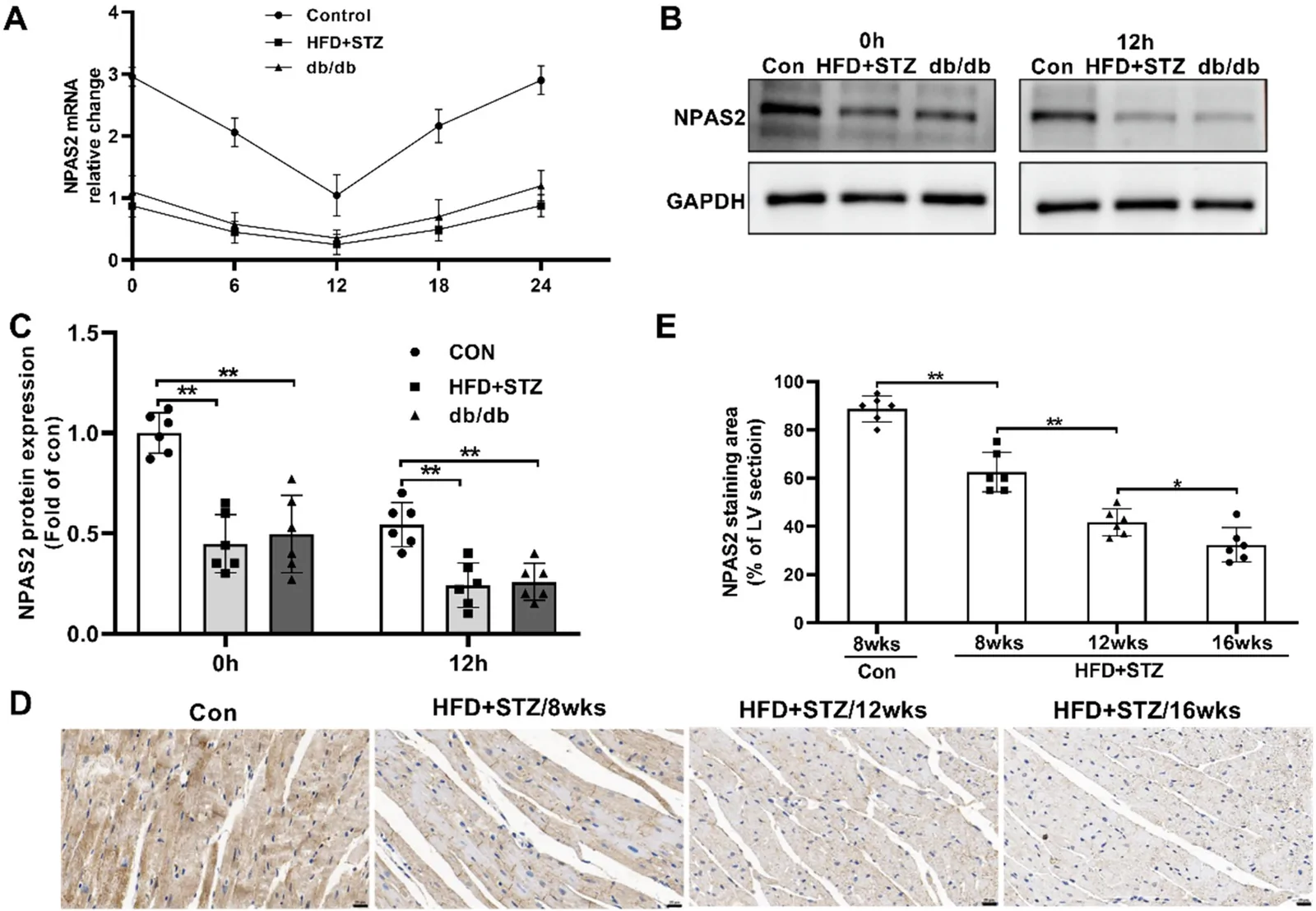
The expression and rhythmicity of NPAS2 were significantly downregulated in T2D mice heart. **(A)** NPAS2 mRNA levels at different ZT points (ZT0, ZT6, ZT12, ZT18, and ZT24). The light/dark cycle is indicated by the white (light phase, ZT0–ZT12) and black (dark phase, ZT12–ZT24) bars. *n* = 6 mice per group per ZT point. **(B,C)** Protein expression level of NPAS2 at ZT0 and ZT12 as determined by western blots. *n* = 6 mice per group per ZT point. **(D,E)** Immunohistochemical staining of NPAS2 protein in left ventricular myocardium. Scale bar: 20 μm.*P < 0.05, **P < 0.01. *n* = 6.

### 3.2 Myocardial-specific deletion of NPAS2 exacerbates cardiac dysfunction and metabolic remodeling in diabetic mice

To elucidate the functional contribution of NPAS2 to the pathogenesis of diabetic cardiomyopathy (DCM), we generated *Npsa2*^cko^ mice and utilized *Npsa2*^flox/flox^ littermates as controls. We subjected both genotypes to a high-fat diet (HFD) combined with streptozotocin (STZ) injection to induce a DCM model. Echocardiographic assessment at 16 weeks of age revealed that (Figures 2A–E), under normal diet (ND) conditions, *Npsa2*^cko^ mice exhibited significantly reduced left ventricular ejection fraction (LVEF%) and fractional shortening (LVFS%) compared to control littermates, while interventricular septum (IVS) thickness remained unchanged. Notably, under the HFD + STZ challenge, *Npsa2*^cko^ mice displayed a further exacerbated decline in cardiac function, accompanied by significant ventricular hypertrophy, as evidenced by increased IVS thickness. Consistently, wheat germ agglutinin (WGA) staining of myocardial sections (Figure 2F) revealed that *Npsa2*^cko^ mice exhibited cardiomyocyte hypertrophy even under normal conditions, which was further aggravated following the induction of diabetes. Furthermore, Masson’s trichrome staining (Figure 2G) demonstrated a marked increase in interstitial fibrosis in the hearts of *Npsa2*^cko^ mice subjected to HFD + STZ compared to the diabetic control group.

**Figure 2:**
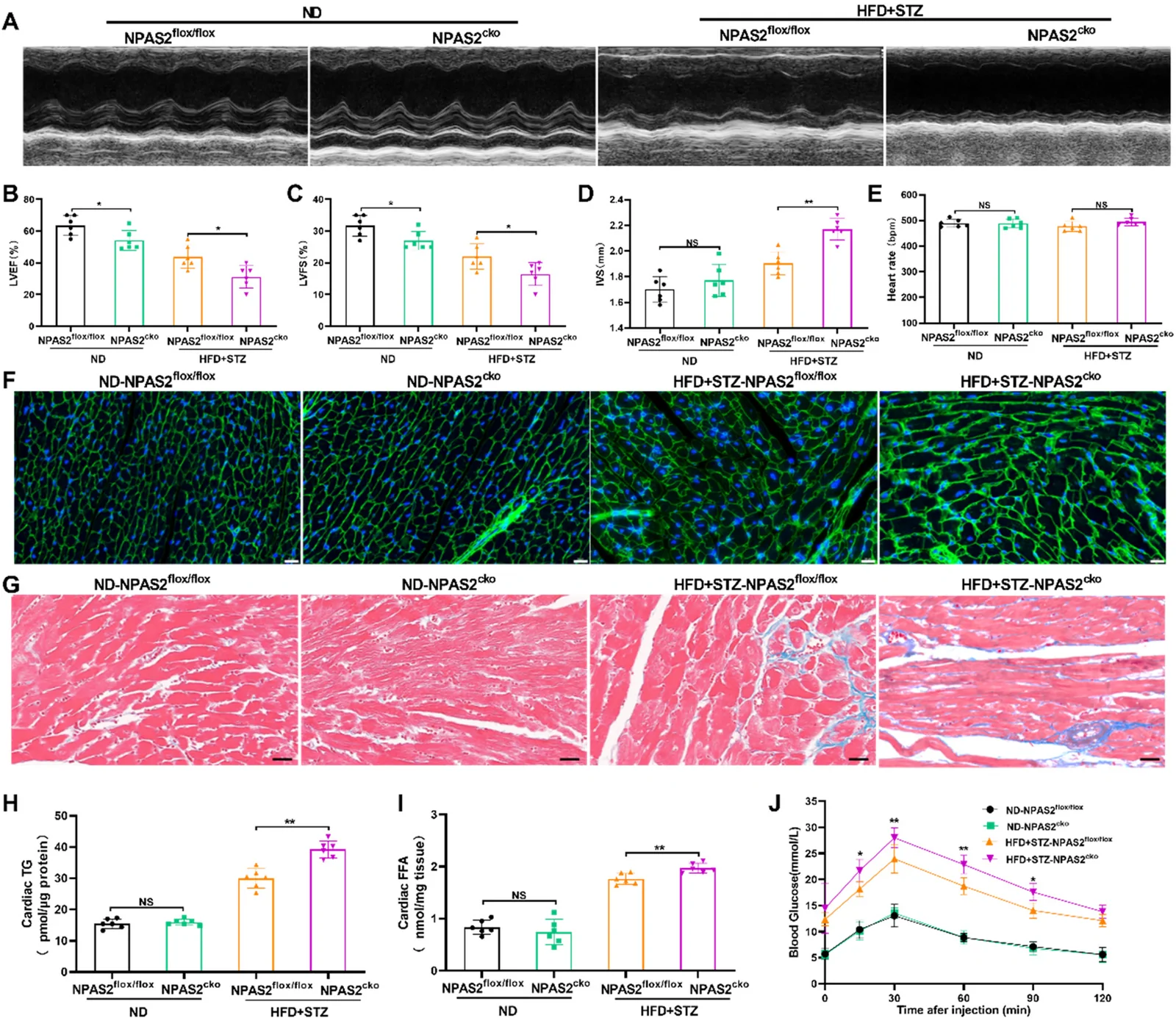
NPAS2 deficiency exacerbates cardiac dysfunction, hypertrophy, fibrosis, and metabolic dysfunction in diabetic cardiomyopathy. **(A)** Representative echocardiographic images of the LV in different 16-week-old mice. **(B,C,D,E)** Echocardiographic assessment of LVEF%**(B)**, LVFS%**(C)**, IVS**(D)**, and heart rate **(E)**, in different mice (*n* = 6, 16 weeks old per group). **(F)** WGA staining of left ventricle myocardium and CSA of cardiomyocytes. Scale bar: 20 μm. **(G)** Masson staining of left ventricle fibrosis and fibrosis area proportion. Scale bar: 20 μm. **(H)** Cardiac triglyceride (TG) content. **(I)** Cardiac free fatty acid (FFA) levels. **(J)** Glucose tolerance tests (GTT). *n* = 6/group, *P < 0.05, **P < 0.01.

Given the critical role of circadian clocks in metabolic regulation (25), we further assessed the impact of *Npsa2* deficiency on cardiac lipid metabolism and systemic glucose homeostasis. While myocardial triglyceride (TG) and free fatty acid (FFA) levels (Figures 2H,I) were comparable between *Npsa2*^cko^ and control mice under a normal diet, their levels were significantly elevated in *Npsa2*^cko^ hearts upon HFD + STZ induction. Similarly, glucose tolerance tests (GTT) (Figure 2J) indicated that although systemic glucose tolerance was unaffected by *Npsa2* deletion under normal dietary conditions, *Npsa2*^cko^ mice challenged with HFD + STZ exhibited significantly impaired glucose tolerance compared to their diabetic controls. These findings collectively demonstrate that myocardial *Npsa2* deficiency aggravates diabetic cardiomyopathy by promoting cardiac maladaptive remodeling and disrupting metabolic homeostasis.

### 3.3 NPAS2 knockdown impairs mitochondrial respiration and promotes metabolic dysfunction in H9c2 cardiomyocytes

To further explore the underlying mechanisms, we utilized H9c2 cardiomyocytes subjected to high-glucose and high-fat (HGHF) conditions to mimic the diabetic microenvironment. We established four experimental groups: Control, siNPAS2, HGHF + Control, and HGHF + siNPAS2. Seahorse XF analysis revealed that NPAS2 knockdown significantly impaired mitochondrial function under basal conditions, as evidenced by reduced spare respiratory capacity, maximal respiration, and ATP production (Figures 3A–E). Notably, while HGHF treatment alone suppressed these respiratory parameters, NPAS2 knockdown further exacerbated these deficits (Figures 3A–E). Consistent with these findings, flow cytometry analysis demonstrated that NPAS2 silencing significantly increased the rate of apoptosis in H9c2 cells, both under normal and HGHF conditions (Figure 3F).

**Figure 3:**
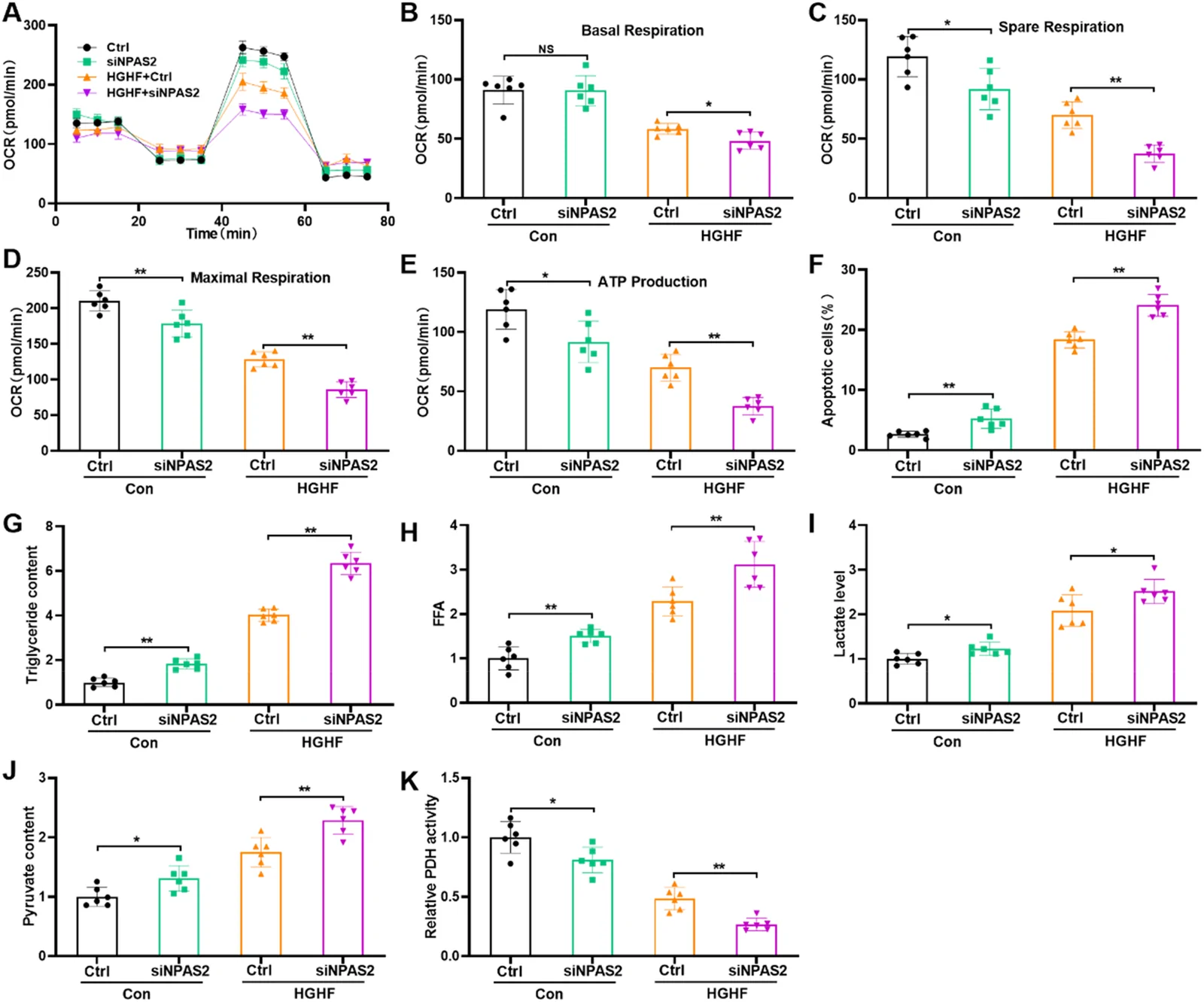
NPAS2 knockdown impairs mitochondrial function, enhances apoptosis, and disrupts metabolic homeostasis in cardiomyocytes under diabetic stress. **(A)** Oxygen consumption rate (OCR). **(B–E)** Analysis and quantification of basal respiration **(B)**, spare respiration **(C)**, maximal respiration **(D)** and ATP production **(E)** in H9c2 myocytes. **(F)** Cell apoptosis rate was determined using flow cytometry. **(G)** Triglyceride content. **(H)** FFA content. **(I)** Lactate level. **(J)** Pyruvate content. **(K)** The activity of pyruvate dehydrogenase (PDH). *P < 0.05, **P < 0.01.

Given the observed mitochondrial dysfunction, we next assessed intracellular lipid and glucose metabolism. NPAS2 knockdown led to a significant accumulation of intracellular triglycerides (TG) and free fatty acids (FFA) regardless of the culture medium (Figures 3G,H). Furthermore, we observed a marked increase in lactate and pyruvate levels in NPAS2-silenced cells compared to their respective controls (Figures 3I,J). To investigate the potential cause of this metabolic shift, we measured the activity of pyruvate dehydrogenase (PDH), a critical enzyme linking glycolysis to the tricarboxylic acid (TCA) cycle. Our results showed that PDH activity was consistently and significantly reduced in the si-NPAS2 groups compared to controls, both under normal and HGHF conditions (Figure 3K). These data suggest that NPAS2 deficiency disrupts mitochondrial bioenergetics and promotes a metabolic shift toward lipid accumulation and impaired glucose oxidation.

### 3.4 Overexpression of NPAS2 attenuates HGHF-induced mitochondrial dysfunction, apoptosis, and metabolic perturbations in cardiomyocytes

To further validate the protective role of NPAS2 and explore its potential as a therapeutic target, we investigated whether restoring NPAS2 expression could mitigate HGHF-induced cellular damage. We utilized an adenoviral vector to overexpress NPAS2 (Ad-NPAS2) in H9c2 cardiomyocytes, with an empty vector (Ad-EV) serving as control. Mitochondrial respiratory function was evaluated using Seahorse XF analysis (Figures 4A–E). Under normal culture conditions, NPAS2 overexpression significantly increased spare respiratory capacity (Figure 4C) compared to Ad-EV control cells, while basal respiration, maximal respiration, and ATP production showed no significant differences (Figures 4A,B, D, E). More importantly, under HGHF conditions, which significantly suppressed these mitochondrial parameters, NPAS2 overexpression effectively restored and robustly upregulated basal respiration, spare respiratory capacity, maximal respiration, and ATP production in H9c2 cells compared to the HGHF + Ad-EV group. Consistent with these improvements in mitochondrial function, cell apoptosis assays (Figure 4F) revealed that while NPAS2 overexpression had no significant effect on apoptosis under normal conditions, it remarkably reduced HGHF-induced cardiomyocyte apoptosis compared to the HGHF + Ad-EV group.

**Figure 4:**
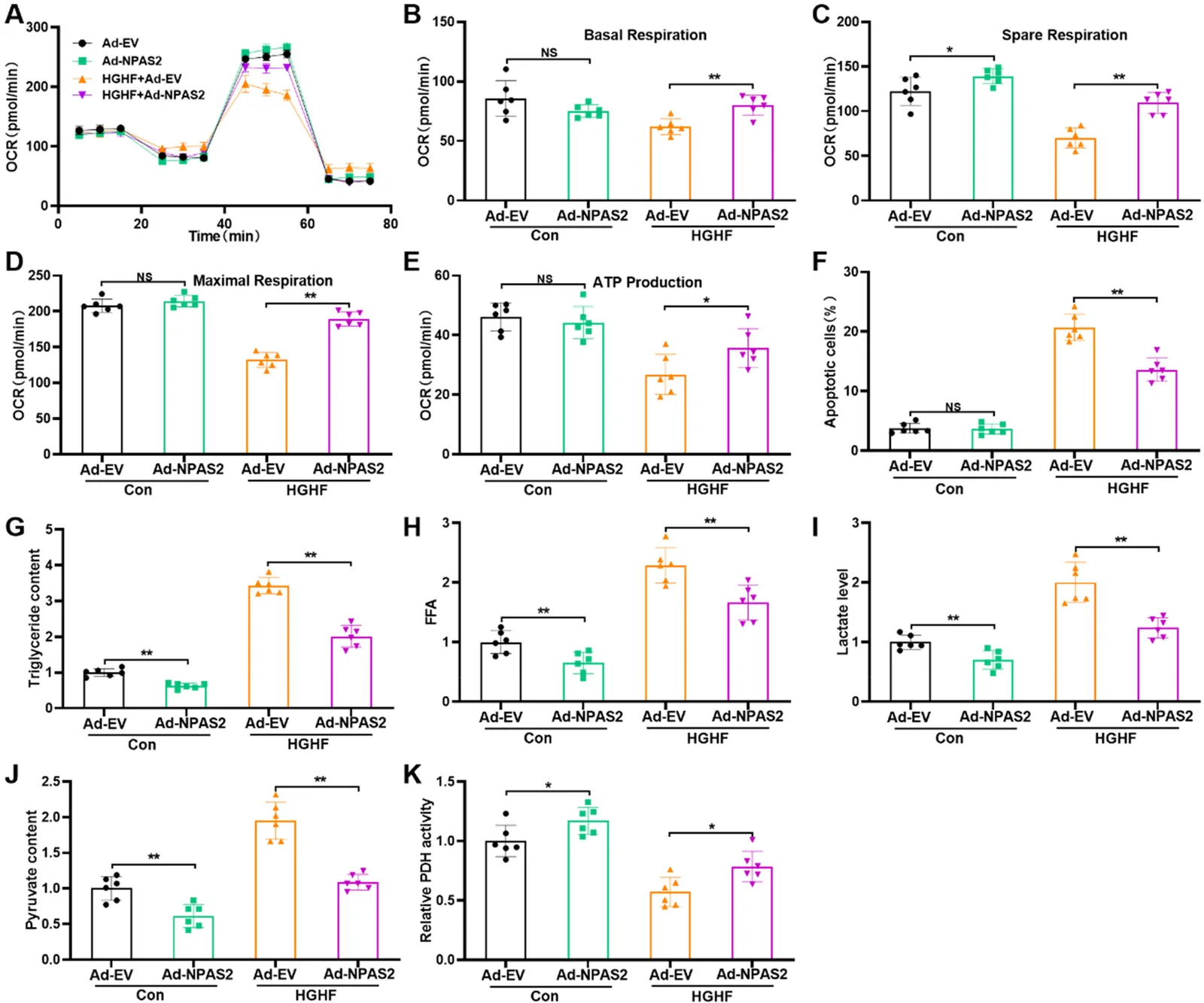
NPAS2 overexpression rescues HGHF-induced mitochondrial dysfunction and metabolic impairment in H9c2 cardiomyocytes. **(A)** Oxygen consumption rate (OCR). **(B–E)** Analysis and quantification of basal respiration **(B)**, spare respiration **(C)**, maximal respiration **(D)** and ATP production **(E)** in H9c2 myocytes. **(F)** Cell apoptosis rate was determined using flow cytometry. **(G)** Triglyceride content. **(H)** FFA content. **(I)** Lactate level. **(J)** Pyruvate content. **(K)** The activity of pyruvate dehydrogenase. *P < 0.05, **P < 0.01.

We then examined the cellular metabolic profiles. NPAS2 overexpression significantly decreased the intracellular accumulation of triglyceride (Figure 4G) and free fatty acid (Figure 4H) in H9c2 cells, under both normal and HGHF culture conditions. Furthermore, analysis of glucose metabolism demonstrated that NPAS2 overexpression significantly lowered the levels of lactate (Figure 4I) and pyruvate (Figure 4J) in H9c2 cells, irrespective of culture conditions. This beneficial effect on carbohydrate metabolism was further supported by the observation that PDH activity, a key enzyme in glucose oxidation, was significantly increased in NPAS2 overexpressing cells under both normal and HGHF conditions (Figure 4K).

Collectively, these *in vitro* overexpression studies strongly support a protective role for NPAS2, demonstrating that its upregulation can effectively counteract HGHF-induced mitochondrial dysfunction, apoptosis, and metabolic dysregulation in cardiomyocytes.

### 3.5 NPAS2 regulates HGHF-induced mitochondrial dysfunction and metabolic stress in a PDH-dependent manner

To determine whether PDH acts as a downstream effector of NPAS2, we performed rescue experiments in H9c2 cells under HGHF conditions. First, we investigated whether PDH overexpression could reverse the deleterious effects of NPAS2 knockdown (siNPAS2). Seahorse XF analysis showed that Ad-PDH significantly restored basal respiration, spare respiratory capacity, maximal respiration, and ATP production in siNPAS2-transfected cells compared to the Ad-EV control (Figures 5A–E). Conversely, we examined whether PDH silencing (siPDH) could abolish the protective effects of NPAS2 overexpression (Ad-NPAS2). As expected, siPDH significantly blunted the improvements in mitochondrial respiration conferred by Ad-NPAS2 (Figures 5A–E).

**Figure 5:**
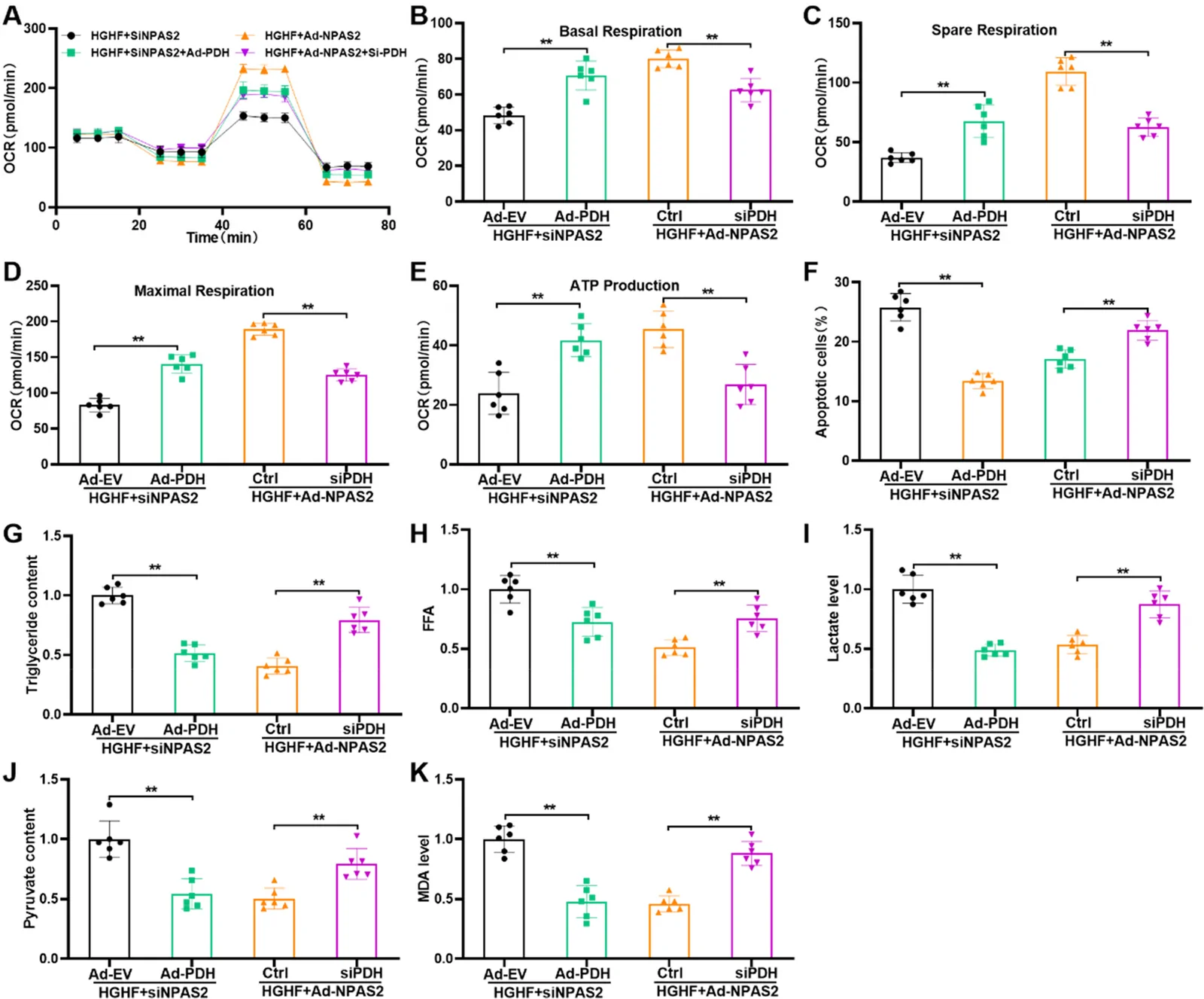
PDH mediates the protective effects of NPAS2 on mitochondrial function and metabolic homeostasis in cardiomyocytes under diabetic stress. **(A)** Oxygen consumption rate (OCR). **(B–E)** Analysis and quantification of basal respiration **(B)**, spare respiration **(C)**, maximal respiration **(D)** and ATP production **(E)** in H9c2 myocytes. **(F)** Cell apoptosis rate was determined using flow cytometry. **(G)** Triglyceride content. **(H)** FFA content. **(I)** Lactate level. **(J)** Pyruvate content. **(K)** MDA level. *P < 0.05, **P < 0.01.

Consistent with these functional findings, flow cytometry demonstrated that PDH overexpression attenuated the increased apoptosis induced by NPAS2 knockdown, whereas PDH silencing exacerbated apoptosis in the presence of NPAS2 overexpression (Figure 5F). Furthermore, metabolic profiling revealed that PDH overexpression significantly reduced the intracellular accumulation of TG and FFA in siNPAS2-treated cells (Figures 5G,H). In contrast, PDH silencing reversed the metabolic benefits of NPAS2 overexpression, leading to increased TG and FFA levels (Figures 5G,H).

Parallel to these changes, PDH overexpression in siNPAS2 cells significantly decreased intracellular lactate and pyruvate levels, while PDH silencing in Ad-NPAS2 cells resulted in a significant accumulation of these metabolites (Figures 5I,J). Finally, we assessed oxidative stress by measuring malondialdehyde (MDA) levels. We found that PDH overexpression mitigated the elevated MDA levels caused by NPAS2 knockdown, whereas PDH silencing abolished the antioxidant effects of NPAS2 overexpression (Figure 5K). Collectively, these data provide compelling evidence that NPAS2 exerts its cardioprotective effects in DCM primarily by maintaining PDH activity and promoting efficient glucose metabolism.

### 3.6 AAV9-mediated NPAS2 overexpression alleviates diabetic cardiomyopathy *in vivo*

To further evaluate the therapeutic potential of NPAS2 in diabetic cardiomyopathy, we performed *in vivo* rescue experiments using adeno-associated virus serotype 9 (AAV9)-mediated NPAS2 overexpression. *Npas2*^cko^ mice and wild-type (WT) controls were subjected to high-fat diet combined with streptozotocin (HFD + STZ) to induce diabetic cardiomyopathy. AAV9-NC (control) or AAV9-NPAS2 was administered via tail vein injection at 9 weeks of age, and all mice were examined at 16 weeks of age. Four groups were included: WT mice subjected to HFD + STZ and injected with control virus (AAV9-NC), WT mice subjected to HFD + STZ and injected with NPAS2-overexpressing virus (AAV9-NPAS2), *Npas2*^cko^ mice subjected to HFD + STZ and injected with control virus (AAV-NC), and *Npas2*^cko^ mice subjected to HFD + STZ and injected with NPAS2-overexpressing virus (AAV-NPAS2).

Echocardiographic analysis (Figures 6A–E) revealed that, under HFD + STZ conditions, AAV9-NPAS2-treated WT mice exhibited significantly increased LVEF% and LVFS% accompanied by reduced IVS, compared with WT mice receiving AAV9-NC. Similarly, in *Npas2*^cko^ mice, AAV9-NPAS2 treatment significantly improved LVEF% and LVFS% and reversed diabetes-induced IVS thickening relative to *Npas2*^cko^ mice injected with AAV9-NC. WGA staining (Figure 6F) demonstrated that NPAS2 overexpression significantly attenuated cardiomyocyte hypertrophy in both WT and *Npas2*^cko^ mice, as evidenced by reduced cross-sectional area compared with their respective AAV9-NC controls. Masson trichrome staining (Figure 6G) further revealed that myocardial interstitial fibrosis was markedly reduced in both genotypes following AAV9-NPAS2 treatment, relative to their corresponding AAV9-NC groups.

**Figure 6:**
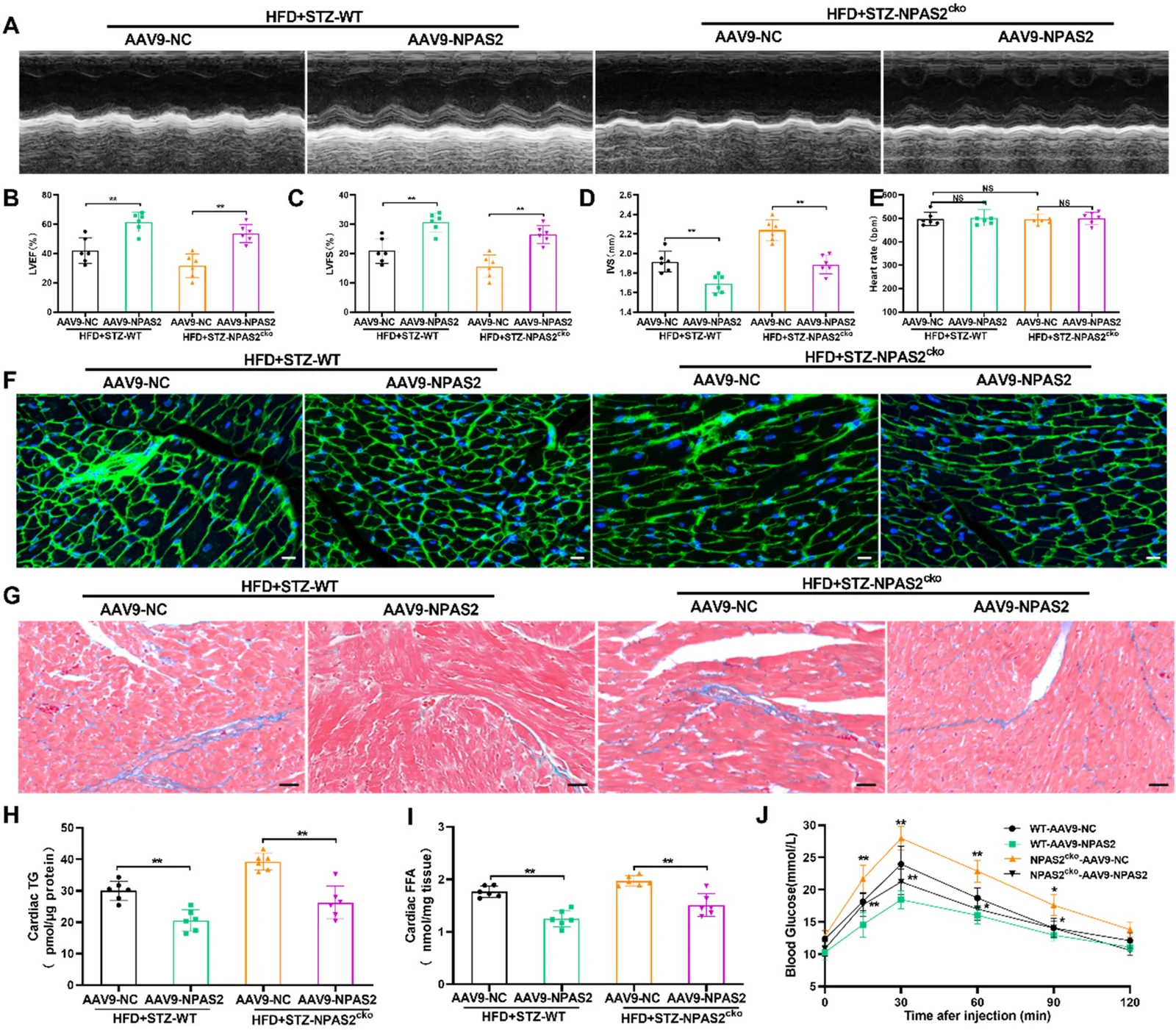
NPAS2 overexpression rescues cardiac dysfunction and metabolic remodeling in diabetic cardiomyopathy. **(A)** Representative echocardiographic images of the LV in different 16-week-old mice. **(B,C,D,E)** Echocardiographic assessment of LVEF%**(B)**, LVFS%**(C)**, IVS**(D)**, and heart rate **(E)**, in diferent mice (*n* = 6, 16 weeks old per group). **(F)** WGA staining of left ventricle myocardium and CSA of cardiomyocytes. Scale bar: 20 μm. **(G)** Masson staining of left ventricle fibrosis and fibrosis area proportion. Scale bar: 20 μm. **(H)** Cardiac TG content. **(I)** Cardiac FFA levels. **(J)** Glucose tolerance tests. *n* = 6/group, *P < 0.05, **P < 0.01.

We next examined cardiac lipid metabolism and systemic glucose tolerance. As shown in Figures 6H,I, cardiac triglyceride (TG) and free fatty acid (FFA) contents were significantly decreased in both WT and *Npas2*^cko^ mice receiving AAV9-NPAS2 compared with their respective AAV9-NC controls. Furthermore, glucose tolerance was significantly improved in AAV9-NPAS2-treated WT and *Npas2*^cko^ mice, as indicated by reduced blood glucose excursion during the glucose tolerance test (Figure 6J).

Collectively, these results demonstrate that AAV9-mediated NPAS2 overexpression effectively alleviates diabetes-induced cardiac contractile dysfunction, myocardial hypertrophy, interstitial fibrosis, and lipid and glucose metabolic disturbances, underscoring the therapeutic potential of NPAS2 in diabetic cardiomyopathy.

## 4 Discussion

DCM represents a serious cardiac complication of diabetes mellitus, characterized by myocardial structural and functional abnormalities that develop independently of coronary artery disease and hypertension (26). The pathophysiology of DCM involves complex interactions among chronic hyperglycemia, lipotoxicity, mitochondrial dysfunction, oxidative stress, and metabolic inflexibility (25, 27). In the present study, we identify NPAS2, a core circadian clock transcription factor, as a critical endogenous protector of the diabetic heart. Our results demonstrate that myocardial NPAS2 expression is significantly suppressed and its circadian rhythm disrupted in diabetic mice across two independent models. Loss of NPAS2 in cardiomyocytes exacerbates diabetes-induced cardiac dysfunction, pathological remodeling, and metabolic dysregulation, whereas restoration of NPAS2 expression, either through adenoviral delivery *in vitro* or AAV9-mediated gene transfer *in vivo*, effectively reverses these abnormalities. Mechanistically, we establish that NPAS2 preserves cardiac metabolic homeostasis by maintaining PDH activity and mitochondrial glucose oxidation capacity, thereby preventing the detrimental shift toward fatty acid utilization that characterizes the diabetic myocardium.

The initial observation that NPAS2 expression declines in the diabetic heart is consistent with a growing body of evidence linking circadian clock disruption to metabolic and cardiovascular disease (28, 29). The circadian molecular network, composed of core clock genes including BMAL1, CLOCK, NPAS2, PERs, and CRYs, governs daily oscillations in myocardial metabolism, contractility, and stress responsiveness (30–32). Disruption of this network has been increasingly recognized as a contributor to the pathogenesis of type 2 diabetes, obesity, and cardiovascular disorders (28, 33). Our finding that NPAS2 expression is progressively downregulated with age in the diabetic myocardium suggests an early and sustained impairment of the myocardial circadian clock, which may precede and contribute to the structural and functional deterioration observed in DCM. This aligns with recent studies demonstrating that NPAS2 expression is dynamically regulated under various pathological stresses, including ischemia/reperfusion injury, where NPAS2 levels decline progressively during reperfusion and overexpression of NPAS2 confers cardioprotection against apoptosis (10). Our study extends these findings by demonstrating, for the first time, that NPAS2 likewise serves a protective role in the diabetic heart.

A particularly novel aspect of our work lies in the identification of PDH as a functional downstream effector of NPAS2 in cardiomyocytes. PDH, the rate-limiting component of the pyruvate dehydrogenase complex, catalyzes the irreversible conversion of pyruvate to acetyl-CoA, thereby linking glycolysis to the tricarboxylic acid cycle and oxidative phosphorylation. In the diabetic heart, PDH activity is markedly reduced, leading to impaired glucose oxidation, increased reliance on fatty acid oxidation, lipotoxicity, and mitochondrial dysfunction (34). Our data show that NPAS2 knockdown in H9c2 cells suppresses PDH activity and accumulates lactate and pyruvate, indicating a block in pyruvate entry into mitochondrial oxidative pathways. Conversely, NPAS2 overexpression restores PDH activity, reduces the accumulation of glycolytic intermediates, and improves mitochondrial respiratory capacity. The rescue experiments further establish a direct causal relationship: PDH overexpression recapitulates the protective effects of NPAS2, while PDH knockdown largely abolishes them. These results strongly suggest that NPAS2-mediated PDH regulation is a central mechanism by which NPAS2 maintains myocardial metabolic flexibility and mitochondrial bioenergetics in the face of diabetic stress.

This functional connection between a circadian clock component and PDH is particularly intriguing in light of previous reports showing that genetic disruption of the circadian machinery impairs PDH activity. For example, muscle-specific deletion of Bmal1 in mice leads to reduced insulin-stimulated glucose uptake and diminished PDH activity, underscoring the importance of the molecular clock in regulating glucose oxidative metabolism (35, 36). Our findings extend this concept by identifying NPAS2 as a specific circadian regulator that acts upstream of PDH in the heart. In addition, NPAS2 has been shown to modulate mitochondrial biogenesis through transcription factors such as PGC-1*α* and to bind promoter regions of genes encoding components of the electron transport chain. Collectively, our data and those of others support a model in which NPAS2 integrates circadian signals with mitochondrial metabolic programs, and where NPAS2 deficiency leads to a breakdown in this integration, ultimately contributing to DCM pathology.

The metabolic consequences of NPAS2 deficiency that we observed in the diabetic heart are consistent with the well-established concept of myocardial metabolic remodeling in DCM. Under normal conditions, the heart derives approximately 60%–70% of its ATP from fatty acid oxidation and the remaining 30%–40% from glucose oxidation (37, 38). In the diabetic heart, this balance is shifted markedly toward fatty acid utilization, resulting in increased lipid accumulation, mitochondrial uncoupling, and generation of reactive oxygen species (38, 39). Our data demonstrate that NPAS2 deficiency exacerbates triglyceride and free fatty acid accumulation in the myocardium, while NPAS2 overexpression reduces lipid content and improves glucose tolerance. These metabolic improvements were accompanied by significant attenuation of myocardial hypertrophy, fibrosis, and contractile dysfunction *in vivo*. This suggests that the NPAS2–PDH pathway plays a critical role in maintaining cardiac metabolic flexibility, and that its disruption is an important contributor to the lipotoxic injury and functional decline observed in DCM.

Importantly, our *in vivo* rescue experiments using AAV9-mediated NPAS2 overexpression demonstrate that restoring NPAS2 expression in the diabetic heart, even in the context of pre-existing NPAS2 deficiency, significantly improves cardiac function, reduces pathological remodeling, and normalizes metabolic parameters. These findings have direct translational relevance, as they indicate that NPAS2 represents a viable therapeutic target not only for prevention, but potentially also for intervention in established diabetic cardiomyopathy. Gene therapy approaches using AAV vectors have advanced substantially in recent years, and the cardiac-specific promoter used in our study ensures efficient and targeted expression of NPAS2 in the myocardium. While significant challenges remain before clinical translation, our results provide a strong rationale for further preclinical development of NPAS2-based therapeutic strategies.

## 4 Limitations

While our study provides evidence supporting a protective role of NPAS2 in diabetic cardiomyopathy, several limitations should be acknowledged. First, although our data support a PDH-dependent function of NPAS2, the precise molecular mechanism by which NPAS2 regulates PDH activity remains incompletely understood. In particular, whether this regulation occurs directly at the transcriptional or post-transcriptional level, or indirectly through upstream modulators such as PDK4, requires further investigation. Second, although H9c2 cells were useful for mechanistic analysis, they do not fully recapitulate the phenotype of adult primary cardiomyocytes; therefore, validation in primary cardiomyocytes or human cell-based models will be important. Third, although AAV9-mediated NPAS2 overexpression produced beneficial effects *in vivo*, the long-term efficacy, safety, and tissue specificity of this strategy remain to be established. In addition, although cardiac structure and function, including myocardial hypertrophy, fibrosis, and echocardiographic parameters, were systematically evaluated in this study, diastolic function remains an aspect that merits further detailed investigation in future work. Finally, further studies are needed to determine whether NPAS2 influences additional metabolic pathways and broader systemic metabolic crosstalk, which may provide deeper insight into its role in diabetic cardiac remodeling.

## 5 Conclusion

In conclusion, we propose that NPAS2 acts as a critical guardian of myocardial metabolic homeostasis in diabetes by sustaining PDH activity and mitochondrial glucose oxidation. The NPAS2–PDH axis represents a previously unrecognized signaling pathway that links circadian clock function to cardiac energy metabolism. Our study not only advances the fundamental understanding of DCM pathophysiology, but also identifies NPAS2 as a promising novel therapeutic target. Restoring NPAS2 expression or enhancing its downstream PDH activity may offer a new avenue for the prevention and treatment of diabetic cardiomyopathy.

## Data Availability

The raw data supporting the conclusions of this article will be made available by the authors, without undue reservation.
